# Primary Intra-operative Embolisation During Urgent Parallel Graft Endovascular Repair in Paravisceral Symptomatic Aortic Pseudoaneurysm^☆^

**DOI:** 10.1016/j.ejvsvf.2024.11.001

**Published:** 2024-11-13

**Authors:** Paolo Spath, Federica Campana, Enrico Gallitto, Chiara Mascoli, Stefania Caputo, Rodolfo Pini, Gianluca Faggioli, Mauro Gargiulo

**Affiliations:** a Vascular Surgery, Department of Medical and Surgical Sciences (DIMEC), University of Bologna, Bologna, Italy; b Vascular Surgery Unit, IRCCS Azienda Ospedaliero-Universitaria di Bologna, Italy; c Vascular Surgery Unit, Hospital «Infermi», AUSL Romagna, Rimini, Italy

**Keywords:** Aortic pseudoaneurysm, Chimney, Coil embolisation, Endovascular repair, Parallel grafts, Penetrating aortic ulcer

## Abstract

**Objective:**

Paravisceral aortic lesions present significant challenges for endovascular treatment. This retrospective analysis of consecutively treated patients from April 2017 to June 2021 aimed to analyse the outcome of primary intra-operative embolisation of aortic complicated pseudoaneurysms and gutter channels during parallel graft (PG) repair of paravisceral symptomatic aortic pseudoaneurysms.

**Methods:**

Patients with symptomatic pseudoaneurysms of the paravisceral aorta treated with PGs using chimney or periscope configurations were included. Thoracic endografts were positioned to exclude the aortic lesions. Coil embolisation of both the lesions and gutter channels was performed after graft deployment and prior to ballooning of the stent grafts. The primary endpoints were technical success (defined as exclusion of the pseudoaneurysm, target visceral vessel [TVV] patency, absence of gutter endoleaks) and clinical success (technical success + resolution of symptoms + absence of major adverse events) at 30 days. Secondary endpoints included overall survival, TVV patency, gutter endoleaks, and freedom from re-interventions during follow up.

**Results:**

Six patients (four women) were treated for pseudoaneurysm rupture (three cases) and symptomatic aortic pseudoaneurysm (three cases) of the paravisceral aorta. The patients' anatomies were unsuitable for off the shelf devices and patients were all deemed to be at prohibitive surgical risk. A total of 15 TVVs were revascularised (comprising three coeliac arteries, five superior mesenteric arteries, and seven renal arteries) using 10 chimney and five periscope PGs. One coeliac artery was occluded. Seventy coils were deployed to embolise both the aortic ruptures and gutter channels. Both technical and clinical success rates were 100%. The median follow up was 17 months (IQR 5, 35), during which time three patients died due to non-aortic related causes. One coeliac artery (6%) was occluded, and no endoleak evidence was found.

**Conclusion:**

Primary intra-operative embolisation during parallel graft endovascular repair of paravisceral symptomatic aortic pseudoaneurysms may be both safe and effective in excluding the pseudoaneurysm when other options are unavailable.

## HIGHLIGHTS

• Paravisceral aortic pseudoaneurysm is a critical scenario for urgent endovascular repair.
• Parallel graft repair by chimney or periscope configurations permits vessel revascularisation.
• Primary intra-operative embolisation managed to exclude aortic ruptures and gutter channels.
• Technical and clinical success was achieved with resolution of symptoms in all treated patients.
• Midterm follow up showed survival with vessel patency and low incidence of re-interventions.

## INTRODUCTION

Paravisceral symptomatic aortic pseudoaneurysms represent a severe condition with a high risk of rupture. The risk of visceral malperfusion and death from haemorrhagic shock is significant.^1^, ^2^, ^3^ This condition may result from spontaneous rupture or the evolution of a penetrating aortic ulcer,^4^, ^5^, ^6^ systemic infectious processes,^7^ or vertebral body erosion due to neoplastic, inflammatory, or infectious diseases.^5^^,^^8^^,^^9^

Standard treatment for lesions at this anatomical location often involves surgical intervention through a thoracophrenic laparotomy approach with supracoeliac clamping.^3^ Alternatively, endovascular techniques using physician modified endografts,^10^^,^^11^ *in situ* laser fenestration endografts, custom made fenestrated endografts,^12^^,^^13^ and branched endografts^14^^,^^15^ can be employed. However, when considering these techniques, technical, customisation, and anatomical feasibility issues must be addressed^16^, ^17^, ^18^, ^19^ and parallel grafts (PGs) may serve as a bailout solution;^20^^,^^21^ there is also an association with a high risk of re-interventions,^22^ particularly resulting from the persistent risk of endoleaks.^23^^,^^24^ In these cases, endoleaks originating from both the gutter channels and the arteries feeding the rupture can contribute to continued bleeding and lesion progression.^25^ Intra-operative coil embolisation of the aneurysm sac or penetrating aortic ulcer, both in the abdominal field and the aortic arch, has been proposed as a viable option to reduce endoleak rates by promoting thrombosis.^4^^,^^26^, ^27^, ^28^, ^29^

This case series aimed to evaluate the effectiveness of intra-operative primary embolisation of aortic lesions and gutter channels in patients presenting with symptomatic paravisceral aortic pseudoaneurysms and who underwent urgent endovascular repair using PGs.

## METHODS

### Patient selection

**Figure 1:**
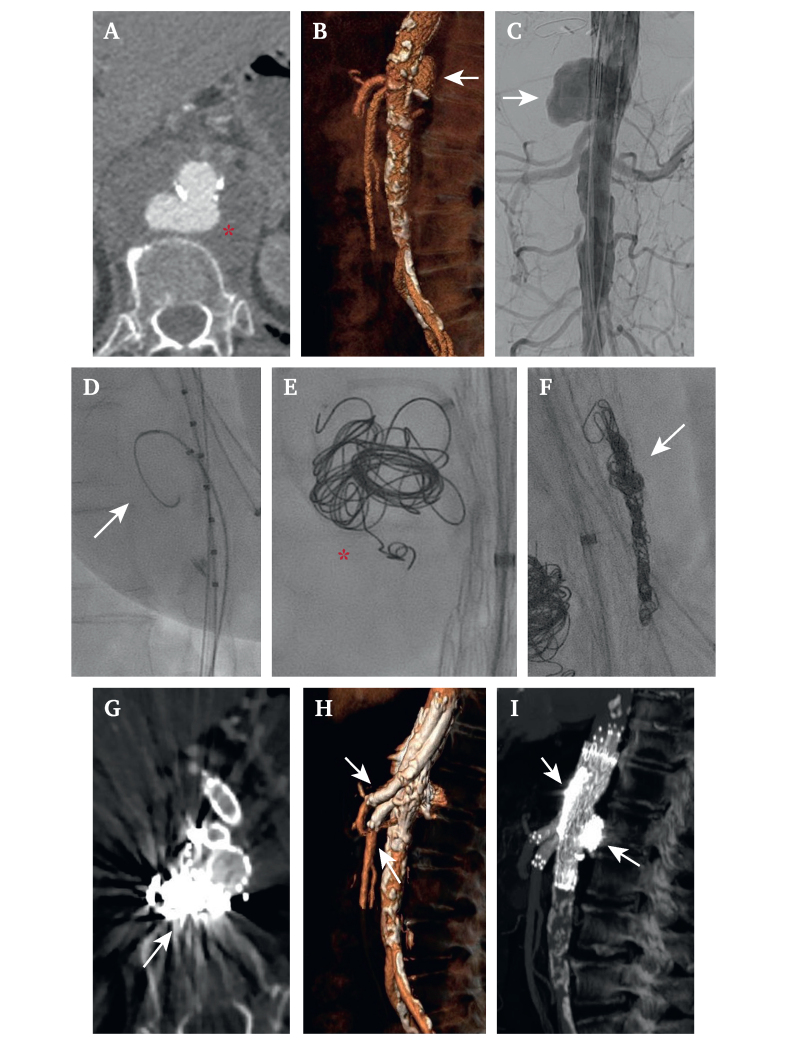
Procedure images. A. Anatomical characteristics of paravisceral aorta associated with symptomatic pseudoaneurysm with contained rupture of the posterior wall with peri-aortic haematoma. B. The pseudoaneurysm involved both zone 6 and zone 7, as shown in the pre-operative volume rendering reconstruction; and C. At the first intra-operative angiogram. D. Procedural step of the embolisation technique: the selected introducer sheath is parked into the pseudoaneurysm before introducing the main stent graft; E. after deployment of thoracic endograft coil embolisation of the pseudoaneurysm is performed; and F. Retrieval of the introducer sheath in the gutter channels and embolisation using detachable coils. G. Post-operative computed tomography angiography result with exclusion of the pseudoaneurysm in the axial projection. H. Details of the chimney revascularisation for the superior mesenteric and coeliac arteries (dialysis patient with planned sacrifice of both renal arteries), and I. Sagittal details of concomitant coil embolisation of the aortic pseudoaneurysm and the gutter channels.

This was a single centre, retrospective, observational study. All consecutive patients presenting with paravisceral symptomatic aortic pseudoaneurysms identified by computed tomography angiogram (CTA)—including those with haemorrhagic shock or signs of rupture, instability, or recurrent symptoms like back or abdominal pain and gastrointestinal disturbances, between April 2017 and June 2021 were included if treated urgently with PG (Fig. 1A–C) after being deemed anatomically and clinically unsuitable for off the shelf devices and open surgery. Due to its retrospective nature, urgent and or bailout types of repair, and unidentifiable information, individual informed experimental consent was waived and no approval was required from the local ethics committee.

### Pre-operative planning and anatomical criteria

Inclusion criteria comprised aortic pseudoaneurysm with a proximal healthy sealing zone above involving one or more target visceral vessels (TVV) including the coeliac artery (CA), superior mesenteric artery (SMA), right renal artery (RRA), and left renal artery (LRA). Both the proximal and distal sealing zones used for excluding the aortic lesion fell between Society for Vascular Surgery zones 4–9.^30^ To guarantee a favourable sealing zone, proximal and distal sealing zones ≥20 mm above and below the lesion were mandatory. Both the aortic arch and the bilateral axillary, brachial, and iliac pathways were evaluated.

### Endovascular procedures with parallel graft

Procedures took place in the Allura Clarity Philips Hybrid operating room under general anaesthesia. Femoral access points were achieved either through surgical arterial exposure or a percutaneous femoral approach using the Proglide system (Abbott Vascular, CA, USA). If upper limb access was necessary, surgical left axillary artery exposure was selected. Systemic heparinisation was administered with a single bolus dose of 5 000 international units of heparin and then activated clotting time guided throughout the procedure. The main body, consistently a thoracic endograft, was advanced with a 260 cm rigid Lunderquist guidewire (Cook Medical, Bloomington, IN, USA) via the chosen femoral access and was placed at the paravisceral aortic level, guided by pre-operative bone markers and fusion imaging. The procedure for cannulation and insertion of PGs has been detailed elsewhere.^31^, ^32^, ^33^

The summarised procedure involves, in the case of a chimney, surgical exposure of the axillary or distal subclavian artery, followed by inserting a standard floppy guidewire into the descending thoracic aorta using a pigtail 5F catheter. On achieving a stable position within the abdominal aorta, an 80 cm long 8/10F Cook Flexor sheath is advanced to the paravisceral aorta. Each TVV is cannulated using a floppy guidewire and a hydrophilic catheter. Following this, a Cook Rosen 260 cm guidewire is inserted distally into the TVV using a glide catheter for guidewire exchange, and then the Flexor sheath is placed within the TVV. A pre-chosen stent graft (either balloon expanding or self expanding) is placed within the TVV but not deployed immediately. This process is repeated for each visceral vessel as needed. For chimney configurations, each stent graft should extend at least 1 cm beyond the proximal end of the previously positioned main body graft to ensure sufficient vessel vascularisation.

In scenarios where a periscope technique was planned beforehand, a similar procedure was performed from the contralateral femoral approach of the main body graft placement for each intended vessel, with the distal end of each stent graft accurately placed at least 1 cm below the distal end of the main body to ensure revascularisation of the vessel by backflow. For pre-planned embolisation of one or more TVVs, a similar approach was employed to cannulate the TVV either from upper or lower limb access, depending on the vessel's orientation, and then occluded with a vascular plug corresponding to the vessel diameter. After all the stent grafts were correctly positioned within the TVVs, they were all simultaneously deployed with the main body.

The embolisation procedure was performed consistently from the femoral access on the side opposite the main body. A 5 F Destination sheath (Terumo Corporation, Tokyo, Japan) or 5F Flexor (Cook Medical) introducer sheath was progressed into the aortic lesion using a 260 cm floppy guidewire and left in place until all endografts were positioned and deployed (Fig. 1D). Following this, the lesion was embolised using both the 45 cm Cook M-Reye coils and Concerto detachable coil system (Medtronic, Minneapolis, MN, USA) via the Terumo microcatheter (Terumo Corporation) (Fig. 1E). The first choice embolisation coil was Cook M-Reye due to the lower incidence of artifacts in post-operative CTA and therefore a better analysis of images, even if without a detachable control system. In cases where extra precise deployment was required, if the leading surgeon's preference, the Concerto detachable coil system was preferred, owing to the possibility of controlling the deployment, with better post-operative images. Post-embolisation of the pseudoaneurysm, the introducer sheath was retracted into the space between the endograft and the aortic wall, and embolisation of the gutter channels was similarly performed in a craniocaudal direction (Fig. 1F). After embolisation, the introducer sheath was removed, and a molding balloon and semi-compliant balloon were used for kissing ballooning of the main body and the stent grafts within the TVVs. Completion angiograms ensured that there were no high flow endoleaks, complete aortic lesion exclusion, and that no TVV stenoses, kinks, or thromboses were present.

### Post-operative follow up

Post-surgery, all patients received a CTA scan within 72 hours (Fig. 1G–I). All patients had a clinical visit with duplex ultrasound within 30 days. If no issues were detected, a second duplex ultrasound at six months and a CTA were scheduled at 12 months. However, if there were indications of graft complications (like types I, II, or III endoleak, aortic lesion enlargement, symptoms of visceral malperfusion, haemodynamic instability, or recurrent back pain), a CTA was conducted with immediate analysis.

### Endpoints and definitions

Primary endpoints were technical and clinical success at 30 days post-operation. Technical success was characterised by exclusion of the paravisceral aortic pseudoaneurysm, no presence of gutter endoleaks, and the absence of TVV stent graft occlusion or severe stenosis post-angiography, no conversion to open surgical repair, and no death within 24 hours. Clinical success combined the criteria for technical success, the resolution of presentation symptoms, and the absence of major clinical adverse events or re-interventions during the first 30 days post-surgery. Instances of cardiac, neurological, and pulmonary morbidity that necessitated additional treatment or extended hospital stay, as well as deteriorating renal function, followed the reporting standards of the Society for Vascular Surgery.^30^ Secondary endpoints considered overall survival, TVV patency, presence of endoleaks, and freedom from re-intervention throughout follow up.

### Statistical analysis

Continuous variables are presented as median values with interquartile range (IQR) where applicable. Categorical data are given as both absolute numbers and percentages. Due to the descriptive nature of this study, no other statistical tests were conducted.

## RESULTS

### Patient selection, anatomical details, and endograft planning

Between April 2017 and June 2021, six patients (two male, four female) underwent urgent PG procedures due to the presence of symptomatic pseudoaneurysms of the paravisceral aorta. Their median age was 80 years (IQR 75, 84). Three cases presented with free aortic rupture, while the other three had symptomatic unruptured pseudoaneurysms (Table 1). The median aortic diameter was 49 mm. The locations of the aortic lesions can be found in Table 2. In the same period, two more patients, one with a rupture above a previous endovascular aneurysm repair (EVAR) treatment and one with a contained ruptured pseudoaneurysm of the paravisceral aorta above a previous open repair, were treated by an off the shelf multibranched endograft (t-branch, Cook Medical).

**Table 1:** Demographics, baseline characteristics and presentation of patients.

Patient	Year of procedure	Age – years	Sex	Operation indication	Main comorbidities	ASA score	Urgent *vs*. delayed procedure	Aortic diameter – mm
1	April 2017	84	Male	Aortic pseudoaneurysm of type IV TAAA in prior treatment of AAA after EVAR	Bifurcated prosthesis EVAR for AAA COPD Myocardial infarction Left nephrectomy for kidney cancer	5	Emergency procedure with haemorrhagic shock	88
2	October 2017	79	Female	Evolution of PAU with symptomatic pseudoaneurysm	Hypertension Prior smoker Bilateral carotid stenosis	3	Delayed procedure with refractory back pain	30
3	June 2020	79	Female	Rapid evolution of PAU with symptomatic pseudoaneurysm	Type II DM Hypertension Sars-COV-2 infection	4	Delayed procedure with refractory back pain	49
4	June 2020	86	Male	Free rupture of pseudoaneurysm evolution of PAU of thoraco-abdominal aorta	Myocardial infarction CKD grade IIIb Atrial fibrillation Recent pneumonia and urinary tract infection	5	Emergency procedure with haemorrhagic shock	63
5	February 2021	81	Female	Rapid evolution of PAU with symptomatic pseudoaneurysm	DM type II COPD CKD grade IV Pulmonary neoplasia	4	Delayed procedure with recurrent back and abdominal pain	58
6	June 2021	65	Female	Free rupture of pseudoaneurysm evolution of PAU of thoraco-abdominal aorta	Type I DM CABG Haemodialysis	4	Emergency procedure with signs of haemorrhagic shock	72

TAAA = thoraco-abdominal aortic aneurysm; AAA = abdominal aortic aneurysm; EVAR = endovascular aortic repair; PAU = penetrating aortic ulcer; COPD = chronic obstructive pulmonary disease; DM = diabetes mellitus; CKD = chronic kidney disease; CABG = coronary artery bypass graft; ASA = American Society of Anaesthesiology.

**Table 2:** Anatomical characteristics of treated aortas and deployed endografts.

Patient	Proximal sealing zone	Proximal sealing diameter – mm	Distal sealing zone	Distal sealing diameter – mm	Thoracic endograft
1	5	28	9	32	Cook Zenith Alpha ZTA-P 40 - 167
2	4	24	6	24	Cook Zenith Alpha ZTA-P 32 - 109
3	5	24	9	22	Gore TGMR 31–26 – 100
4	5	25	9	20	Gore TGM 34–34 - 150
5	7	22	9	22	Cook Zenith Alpha ZTA-P 36 - 161
6	5	21	8	21	Gore TGMR 31–26 - 100

### Intervention details

Bilateral femoral surgical access was employed in all cases. A left axillary cutdown was performed in four of the six patients, employing single or multiple punctures to revascularise the TVVs. Patient one underwent a bilateral axillary approach: the CA was revascularised from the right side, while the RRA and SMA were addressed from the left. Patient four had a right axillary cutdown due to a chronic left subclavian artery occlusion. A single thoracic endograft was deployed in all six cases (Table 2).

A total of 15 TVVs were revascularised: three (20%) CA, five (33%) SMA, four (27%) RRA, and three (20%) LRA. Notably:

- Patient one had a chronic LRA occlusion
- Patient two underwent revascularisation of only the CA
- Patient three had the CA embolised after confirming revascularisation from SMA collaterals
- Patient four had a chronic CA occlusion
- Patient five had a CA well above the pseudoaneurysm, so the endovascular procedure did not involve this artery
- Patient six, being on chronic dialysis, had both renal arteries unaddressed.

Chimney configurations from above were the most common revascularisation method, applied in 10 (63%) of the TVVs. Periscope configurations from below were employed in five (31%) TVVs. After observing vascularisation compensation of both the hepatic and splenic arteries from the SMA in patient three, CA embolisation was conducted using a vascular plug, streamlining the procedure without perfusion issues.

Seventeen stent grafts were used in total, with an additional 12 bare metal stents deployed. Specifically, 62 Cook M-Reye coils filled the aortic pseudoaneurysms and the wider gutter channels, and eight Concerto detachable coils embolised the gutter channels in two cases with limited space between the channel and endograft end (Table 3). All patients were taken to the intensive care unit after the procedure.

**Table 3:** Details of procedural parallel graft and embolisation.

Patient	Target visceral vessels	Parallel graft technique	Stent graft used	Need for bare metal stent	Introducer sheaths for embolisation	Embolisation coil details	Any adjunctive procedures
1	RRA	Chimney	Viabahn 7x100 Viabahn 7x50	Protégé 8x100	Terumo Destination 5 F 45 cm	Total 10 coils 10 Cook M-Reye	
	SMA	Chimney	Viabahn 9x100	Luminexx 9x100			
	CA	Chimney	Viabahn 11x100	Luminexx 12x80 Luminexx 12x60			
2	CA	Chimney	Viabahn 7x150	Protégé 7100 Protégé 7x80	Terumo Destination 5 F 45 cm	Total 5 coils 5 Cook M-Reye	Left Iliac artery PTA (Armada 7x80)
3	CA	Embolisation	Amplatzer Plug Trilobe 14 mm	/	Cook Flexor 5 F 90 cm	Total 9 coils 6 Cook M-Reye 3 Concerto	PTA (Armada 8x20) of SMA to solve type Ic endoleak
	SMA	Chimney	VBX 7 79	None			
	LRA	Periscope	VBX 5x79	None			
	RRA	Periscope	VBX 6x79	None			
4	SMA	Chimney	VBX 7x79 VBX 7x59	None	Cook Flexor 5 F 90 cm	Total 28 coils 28 Cook M-Reye	
	RRA	Periscope	VBX 5x79	None			
	LRA	Periscope	VBX 5x79	Protégé 6x100			
5	SMA	Chimney	Vibahn 7x100	Protégé 8x100	Terumo Destination 5 F 45 cm	Total 13 coils 13 Cook M-Reye	Destino 7 Fr 105 cm steerable sheath Right iliac artery (Armada 7x60) Left femoral artery endarterectomy
	RRA	Chimney	Viabahn 6x100	Protégé 7x100			
	LRA	Periscope	Viabahn 6x150	Protégé 7x100			
6	SMA	Chimney	Viabahn 8x100	Zilver 8x100	Cook Flexor 5 F 90 cm	Total 5 coils 5 Concerto	
	CA	Chimney	Viabahn 7x100	Protégé 7x100			
Total	15 TVV + (1 CA embolised)	10 chimney 5 periscope 1 Emboliz	17 stent graft 1 vascular plug	12 Bare metal stents needed	3 x Terumo destination 5 F 45 cm 3 x Cook Flexor 5 F 90 cm	Total 70 coils 62 Cook M-reye 8 Concerto	5 adjunctive procedures needed

RRA = right renal artery; SMA = superior mesenteric artery; CA = coeliac artery; LRA = left renal artery; PTA = percutaneous angioplasty; TVV = target visceral vessel.

### Early results

Intra-operative angiograms confirmed technical success in all cases. No deaths or re-interventions occurred either during the hospital stay or within the first 30 days. The median hospital stay was 12 days (IQR 7, 23) (Table 4). Patient one, who was treated for a type IV thoraco-abdominal aortic aneurysm rupture following a prior EVAR presenting with haemorrhagic shock symptoms, demonstrated impaired renal function (2.06 mg/mL creatinine, eGFR 28 mL/min); this issue resolved spontaneously with rehydration. Pre-discharge CTA scans revealed no endoleaks or gutter endoleaks in any patient. Patients 1, 4, and 6, who were on anticoagulants for cardiological reasons, were discharged with the addition of lifelong single antiplatelet therapy; the remainder had double antiplatelet therapy for six months, followed by single therapy thereafter.

**Table 4:** Early and follow up results.

Patient	Hospital time – days	Endoleak discharge	Follow up events	Action required	Freedom follow up event – months	Patient status	Cause of death	Follow up time – months	Aortic lesion
1	25	None	None	\	\	Died	Congestive acute heart failure	8	Stable, no EL
2	6	None	Occlusion CA	None	21	Alive	\	71	Reduced, no EL
3	8	None	None	\	\	Alive	\	39	Reduced, lost to follow up
4	23	None	Endocarditis	Medical therapy	3	Died	Endocarditis/sepsis	3	Stable, no EL
5	12	None	None	\	\	Died	Covid-19 pneumonia	4	Stable, no EL
6	13	None	Endograft infection	Antibiotic therapy	2	Alive	\	26	Stable, no EL

CA = coeliac artery; EL = endoleak.

### Follow up results

The median follow up duration was 17 months (IQR 5, 35) (Table 4). Of the initial six patients, three died during the first year of follow up at three, four, and eight months, respectively; however, none of these deaths were aorta related. Patient 2 experienced a unique TVV related event: CA occlusion after 21 months. No further interventions were deemed necessary due to the absence of symptoms and adequate collateralisation. No additional interventions, endoleaks, or gutters were identified during follow up. Patient 6, who was treated for an aortic rupture and previously hospitalised with infection symptoms, was re-admitted two months post-discharge. Combined CTA and positron emission tomography revealed a successful endovascular procedure outcome, devoid of endoleaks, but indicated an infectious process involving the endograft and spine due to spondylitis. Owing to the high risks associated with an open surgical conversion and graft excision, prolonged antibiotic treatment was prescribed. The patient remains alive and well after 26 months.

## DISCUSSION

This case series highlights the utility of primary embolisation of both the aortic lesion and potential gutter channels, in the context of paravisceral symptomatic aortic pseudoaneurysm, as a bail out procedure when both standard endovascular options are unavailable and open surgical repair is not feasible. The treatment employed parallel graft techniques together with primary pre-emptive embolisation to mitigate the risk of both early and late endoleaks, and consequently preventing lesion progression.

Despite the high reported mortality and morbidity rates, 13% and 55%, respectively, open repair remains the gold standard for addressing symptomatic pseudoaneurysms of the paravisceral aorta.^3^ However, endovascular procedures have come to the fore, especially for urgent and high risk patients, demonstrating safer outcomes when anatomically and technically feasible.^5^^,^^14^^,^^34^

Parallel graft techniques, including the chimney and periscope procedures,^31^^,^^32^^,^^35^ initially emerged for TVV revascularisation in elective repair scenarios like thoraco-abdominal aortic aneurysms and juxta and pararenal aneurysms. Today, they might be recommended in urgent scenarios when other treatments are impractical due to various challenges (Recommendation 129, Class IIa, Level C),^21^ when the risk of open surgery is unacceptable, when there is no time for tailored fenestrated and branched EVAR,^16^ when a physician modified endograft is impossible in a case of the patient being too unstable for re-operation of the fenestrated graft, or lack of experience of the centre.^11^ Endoleaks are still a significant concern, with reported rates ranging 13–30%.^24^^,^^36^ Most of these are found in the gutter channels. Such complications necessitate interventions like embolisation or surgical conversion.^22^^,^^23^

In this series, the decision to use PG was grounded in clinical and anatomical factors. Patients were treated urgently, typically within the first 24 hours from the admission. Given the pre-operative risk factors, open surgical repair with thoracophrenic laparotomy was considered prohibitive. Considering repair with a commercially available off the shelf multibranched endograft in the centre (t-Branch, Cook Medical),^14^ the narrow aortic diameter at the level of the visceral arteries was a major anatomical contraindication.^18^^,^^37^ As recently published, a paravisceral aorta t-Branch needs a diameter of 25 mm,^19^ reaching an overall feasibility in 48% of cases;^17^ that minimum required diameter was not present in any of the current cases, except patient 1, with high risk of failure of branch deployment. For patient 1, the previous EVAR resulted in a distal main body that was too short and expansive to accommodate a proximal t-Branch, necessitating a 40 mm thoracic EVAR instead. At that time, sequential deployment was still not practiced in the clinical setting, published in 2021,^38^^,^^39^ while this can often now be used in similar situations.^40^ However, parallel graft may still represent a possible option if a t-Branch is unavailable or in order to reduce healthy aortic coverage.^17^

Typically, this centre opts for the left axillary approach to reduce cerebral embolism risks, but a right sided approach is viable under certain circumstances.^41^ The main advantage of axillary artery exposure is the availability of a large diameter vessel, permitting parallel puncture for insertion of multiple parallel grafts. The aortic exclusion was uniformly executed using short available thoracic modules, potentially minimising risk of spinal cord ischaemia.^17^^,^^42^ Importantly, there were no neurological issues.

The embolisation strategy aimed to prevent continued aortic bleeding and mitigate the gutter endoleak impact. This tactic mirrors standard practices described for the infrarenal aorta.^4^^,^^26^^,^^27^^,^^29^ Several precautions were adhered to, to ensure smooth execution of the embolisation:

1) the introducer sheath chosen for embolisation should be parked before inserting the main body into the lesion
2) during the manoeuvres, operators must avoid inadvertent extraction of the introducer from the space between the endograft and the aortic wall
3) embolisation must be performed before completion of kissing ballooning of the endograft and TVV stent grafts to reduce the risk of displacement of the endograft material during retraction of the introducer This series used two types of coils: the not controlled detachment Cook M-Reye (Cook Medical., Bloomington, IN, USA) and Concerto detachable coil system (Medtronic Inc., Santa Rosa, CA, USA). The M-Reye coils were preferred for aortic lesions and larger areas to be embolised, whereas the Concerto coils were chosen for selected embolisation areas such as tight and short portions of the gutter channels and were inserted via dedicated microcatheters. In all cases, the planned embolisation procedure was fully completed without complications, inadvertent coil release, or distal embolisation.

The initial results are promising: 100% technical success, no re-intervention due to endoleaks, and no early deaths. This aligns with literature reports on the chimney and periscope technique.^31^^,^^33^^,^^35^ Follow up found that whilst half of the patients died within a year post-procedure, none of these deaths were aorta related, testifying to a fragile population that always needs to be considered at the time of repair. One patient encountered a TVV related complication, but no intervention was required due to collateral perfusion. Moreover, the main finding was that no patient developed new endoleaks during the follow up period.

This study had some limitations. It was retrospective and based on a single centre's experience, making its results non-generalisable. At the same time, the specific aetiology of these lesions, especially for some patients like patient 6, were difficult to rule out since, in many cases, the extensive blood tests and positron emission tomograms were inconclusive due to the presence of a highly inflammatory process at the level of the aortic region. In addition, the endovascular management did not permit direct sampling of the aortic lesion for histological and laboratory workup. The case count and follow up duration were limited, and the absence of a control group further limited the conclusions.

### Conclusions

Primary intra-operative embolisation for paravisceral symptomatic aortic pseudoaneurysms treated by urgent or emergency endovascular repair using parallel grafts seems to be effective and safe when other standard endovascular options are unavailable or not feasible and open surgical repair is contraindicated. Technical and clinical outcomes, even if in a small preliminary series that needs to be further expanded to gain validation, appear to be satisfactory, with the embolisation process reducing early endoleaks and ensuring sealing of the pseudoaneurysm.

## Conflict of Interest

M.G., G.F, and E.G. are consultants for Cook Medical for fenestrated and branched endovascular aneurysm repair. The other authors declare that they have no competing interests.

## Funding

None.
